# In this issue: Volume 115, Issue 10, October 2024

**DOI:** 10.1111/cas.16358

**Published:** 2024-10-03

## Tumor‐derived mitochondrial formyl peptides suppress tumor immunity through modification of the tumor microenvironment

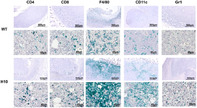

Cancer immunotherapy requires a thorough understanding of the tumor microenvironment (TME)—the ecosystem surrounding a tumor. TMEs are composed of various cellular components, which travel through blood to reach the tumor site. These components are recruited by tumor cell‐derived proteins called ‘chemoattractants’ and are thus crucial for TME formation. *N*‐formylpeptides are chemoattractants that have been well studied in bacteria. These peptides interact with formyl peptide receptors to recruit a variety of cells. In mammalian cells, mitochondrial *N*‐formylpeptides are released from dead and damaged cells to extracellular spaces, and they play an important role in triggering inflammation after trauma or surgeries. However, the role of these peptides in cancer has remained unclear.

This study investigated the impact of tumor cell‐derived mitochondrial *N*‐formylpeptides on cancer growth. Using CRISPR/Cas9 gene editing, the gene encoding mitochondrial methionyl‐tRNA formyltransferase (MTFMT), an enzyme essential for mitochondrial *N*‐formylpeptide production, was inactivated in E.G7‐OVA mouse lymphoma cells. These modified cells were then implanted into C57BL/6 mice to observe tumor growth.

Results showed that tumors formed by MTFMT‐knockout cells exhibited significantly slower growth compared to those formed by unmodified (wild‐type) E.G7‐OVA cells. This finding suggests that mitochondrial *N*‐formylpeptides contribute to accelerated tumor growth. The TME of the MTFMT‐knockout tumors contained fewer myeloid‐derived suppressor cells, which typically help tumors evade the immune system, and more cytotoxic T‐lymphocytes, which target and destroy tumor cells. This indicates that mitochondrial *N*‐formylpeptides may promote cancer progression by modifying the TME to suppress the body's natural anti‐tumor immune response.

These findings highlight mitochondrial *N*‐formylpeptides as potential targets for cancer immunotherapy.

https://onlinelibrary.wiley.com/doi/10.1111/cas.16266

## Plasma cell‐free DNA methylome‐based liquid biopsy for accurate gastric cancer detection

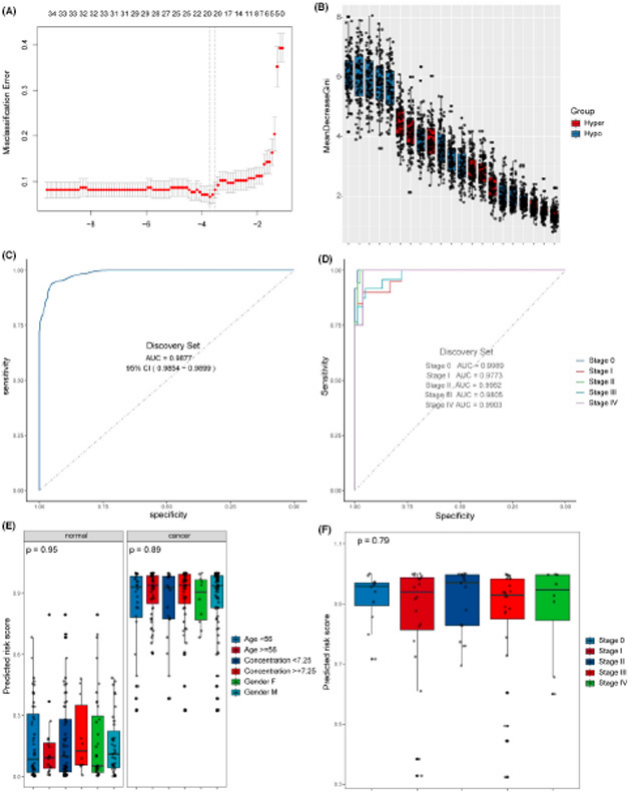

Gastric cancer is one of the most common cancers worldwide and has a high mortality rate. Early detection can potentially reduce death rates associated with this disease, but many cases are diagnosed at a later stage, resulting in poorer outcomes. Currently early detection methods, such as endoscopy and serum markers, can cause discomfort, risk of complications, or exhibit low efficacy. This underscores the need for a more accurate and safer early detection approach for gastric cancer.

Cell‐free DNA (cfDNA), which arises from the death of both normal and tumor cells, is commonly found in the bloodstream. Genetic variations, represented by methylation changes in tumor‐derived cfDNA, can serve as a potential biomarker for early cancer detection.

In this study researchers used an advanced technique called cell‐free methylated DNA immunoprecipitation and high‐throughput sequencing (cfMeDIP‐seq) method to identify gastric cancer‐specific biomarkers from cfDNA methylation. Unlike the commonly used bisulfite sequencing, cfMeDIP‐seq requires only a small amount of DNA and does not damage the original DNA sequence, making it ideal for samples with low DNA quantities.

The researchers analyzed plasma samples from 150 patients with gastric cancer and 100 healthy controls to conduct a genome‐wide methylation profiling of the extracted cfDNA. They identified 21 differentially methylated regions (DMRs) between the gastric cancer and control groups. Using these DMRs, they developed a random forest model for detecting gastric cancer. The model was trained using 80% of the samples and validated with the remaining 20%.

The authors found that the model accurately detected gastric cancer with a sensitivity of 93.90% and specificity of 95.15% in the training samples, and 88.38% and 94.23% in the validation samples, respectively. Furthermore, the model's accuracy remained consistent across various clinical characteristics, including age, gender, and cfDNA concentration, indicating its broad applicability.

These findings highlight the efficacy and accuracy of cfDNA‐derived methylation biomarkers in the early identification of gastric cancer, potentially leading to the development of improved diagnostic and prognostic tools for gastric cancer.

https://onlinelibrary.wiley.com/doi/10.1111/cas.16284

## Delta‐6 desaturase FADS2 is a tumor‐promoting factor in cholangiocarcinoma

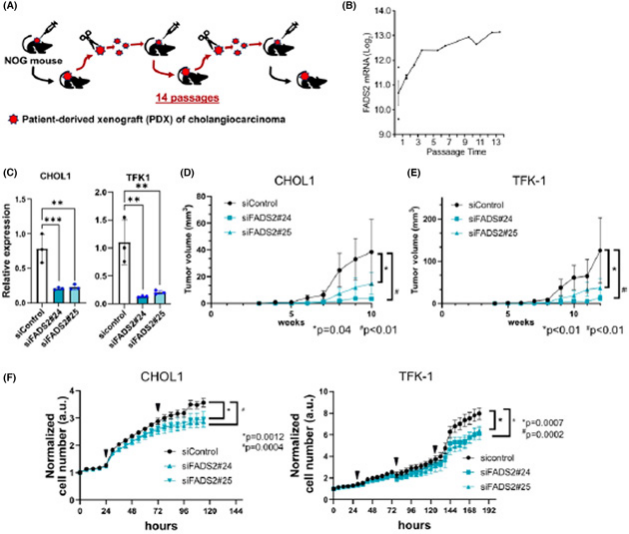

Cholangiocarcinoma (CCA) is a rare but aggressive form of cancer that originates in the bile ducts. This tumor is composed of various types of cells, and its uncontrolled growth is supported by altered various cellular and biochemical pathways. In the recent years, the accumulation of fat molecules by specific enzymes has been found to be involved in the spread of cancer to other organ systems. However, the specific role of fatty acids and their regulatory enzymes in CCA has remained unclear.

To address this, researchers developed a specialized mouse model to study bile duct cancer cells derived from patients. They focused on a particular enzyme called fatty acid desaturase 2 (FADS2), which is involved in converting dietary fats into essential fatty acids. Initially, gene expression studies revealed that *FADS2* was highly expressed in these cancer cells but not in the surrounding stromal cells. Silencing the *FADS2* gene resulted in decreased proliferation of the patient‐derived cancer cells and in vivo tumorigenicity.

Further validation using a FADS2 inhibitor demonstrated that blocking this enzyme reduced migration of cancer cells and their ability to form spheres, both indicators of aggressive cancer behaviour. Additionally, FADS2 inhibition promoted apoptosis and ferroptosis, the latter of which is a type of cell death associated with iron and lipid metabolism.

They also examined the different types of fat molecules present in the patient‐derived cancer cells to understand how FADS2 affects fatty acid content. Reducing FADS2 activity was associated with lower lipid content, suggesting that it plays a key role in regulating fat metabolism. Furthermore, the study assessed FADS2's impact on energy metabolism through the beta‐oxidation pathway, which breaks down fat molecules to produce energy. Inhibition of FADS2 resulted in decreased beta‐oxidation, indicating that it is crucial for energy production in cancer cells, impacting cancer cell malignancy.

Overall, the study provides valuable insight into the complex molecular mechanisms of FADS2 and its role in regulating cancer cell growth and spread. Targeting FADS2 could be a promising strategy for developing new therapeutic approaches to improve treatment outcomes for patients with bile duct cancer.

https://doi.org/10.1111/cas.16306

